# Genetic Variants of DMBT1 and SFTPD and Disease Severity in Paediatric Inflammatory Bowel Disease—A Polish Population-Based Study

**DOI:** 10.3390/children8110946

**Published:** 2021-10-21

**Authors:** Aleksandra Glapa-Nowak, Mariusz Szczepanik, Aleksandra Banaszkiewicz, Barbara Iwańczak, Jarosław Kwiecień, Anna Szaflarska-Popławska, Urszula Grzybowska-Chlebowczyk, Marcin Osiecki, Jarosław Kierkuś, Marcin Banasiuk, Tomasz Banasiewicz, Jens Madsen, Jarosław Walkowiak

**Affiliations:** 1 Department of Pediatric Gastroenterology and Metabolic Diseases, Poznań University of Medical Sciences, 60-572 Poznan, Poland; glapa@ump.edu.pl (A.G.-N.); mszczepanik@ump.edu.pl (M.S.); 2 Department of Pediatric Gastroenterology and Nutrition, Medical University of Warsaw, 02-091 Warszawa, Poland; abanaszkiewicz@wum.edu.pl (A.B.); marcin.banasiuk@wum.edu.pl (M.B.); 3 Department and Clinic of Pediatrics, Gastroenterology and Nutrition, Wroclaw Medical University, 50-367 Wrocław, Poland; barbara@iwanczak.com; 4 Department of Pediatrics, Faculty of Medical Sciences in Zabrze, Medical University of Silesia, 40-055 Katowice, Poland; jkwiecien@sum.edu.pl; 5 Department of Pediatric Endoscopy and Gastrointestinal Function Testing, Collegium Medicum in Bydgoszcz, Nicolaus Copernicus University in Toruń, 85-067 Bydgoszcz, Poland; anna.szaflarska@cm.umk.pl; 6 Department of Pediatrics, Faculty of Medical Sciences, Medical University of Silesia, 40-752 Katowice, Poland; uchlebowczyk@sum.edu.pl; 7 The Department of Gastroenterology, Hepatology, Feeding Disorders and Paediatrics, The Children’s Memorial Health Institute, 04-730 Warsaw, Poland; M.Osiecki@IPCZD.Pl (M.O.); j.kierkus@med-net.pl (J.K.); 8 Chair and Department of General Surgery, Gastroenterological Surgical Oncology and Plastic Surgery, Poznań University of Medical Sciences, 60-572 Poznan, Poland; tbanasie@ump.edu.pl; 9 The Elizabeth Garrett Anderson Institute for Women’s Health, Faculty of Population Health Sciences, University College London, London 84-86, UK; jens.madsen@ucl.ac.uk

**Keywords:** Crohn’s disease, exacerbation, hospitalisation, immunosuppression, ulcerative colitis, polymorphism, relapse

## Abstract

Deleted in malignant brain tumours 1 protein (DMBT1) and surfactant protein D (SFTPD) are antimicrobial peptides previously linked to inflammatory bowel disease (IBD) susceptibility. This study attempts to link the most potential IBD-associated polymorphisms in DMBT1 and SFTPD with the disease severity in children. A total of 406 IBD patients (Crohn’s disease (CD) *n* = 214 and ulcerative colitis (UC) *n* = 192) were genotyped using hydrolysis probe assay. Clinical expression was described by disease activity scales, albumin and C-reactive protein levels, localisation and behaviour (Paris classification), systemic steroid, immunosuppressive, biological, and surgical treatment, number of exacerbation-caused hospitalisations, relapses and nutritional status. IBD patients with the risk genotype (AA) in DMBT1 rs2981804 had more frequent biological treatment (AA: vs. AG/GG; *p* = 0.012), concomitant diseases (AA vs. AG vs. GG; *p* = 0.015) and cutaneous manifestations (AA vs. AG/GG, *p* = 0.008). In UC, rs2981804 genotypes might be linked with albumin concentrations at diagnosis (AA vs. AG vs. GG; *p* = 0.009). In CD, DMBT1 rs2981745 was significantly associated with the number of severe relapses per year of disease (*p* = 0.020) and time-to-immunosuppression (*p* = 0.045). SFTPD was seemingly found to be associated with age at first immunosuppression in IBD (CC vs. CT vs. TT; *p* = 0.048). In conclusion, selected polymorphisms of DMBT1 and SFTPD might be associated with some disease severity measures in children with IBD. However, the magnitude of associations and their clinical relevance might be minor.

## 1. Introduction

Inflammatory bowel disease (IBD) represents a complex multifactorial condition of growing incidence in newly industrialised countries [1]. Available data suggest that a pivotal role is played by predisposing genetic alterations as well as environmental factors in the complex pathogenesis of IBD. Identification of disease-specific and course-specific genetic markers could help to delineate biological pathways involved in disease development and treatment [2].

To date, it has already been proved that some of the genetic markers serve as a prognostic indicator for certain phenotypes [3]. These include the most studied variants in susceptibility gene NOD2 (CARD15), which is associated with small bowel disease, stricturing disease [4] and early surgery in Crohn’s disease (CD) [4,5,6,7,8,9,10]. Despite the plethora of studies in this field, the clinical utility of this gene’s polymorphisms remains undetermined, with studies suggesting conflicting results [11,12]. Several other genetic variants have been reported, such as the variant in the transcription factor FOXO3P associated with more severe CD assessed by medication escalation [13]. Mutations in the gene coding of the autophagy-related protein 16-1 are associated with stricturing disease and perianal involvement in CD [10]. Multidrug resistance 1 gene, which encodes a protein transporting substrates across the cell membrane, has been reported to be associated with more severe IBD and resistance to treatment [14]. Gene polymorphisms in TNF-α receptors have been reported with response failure to infliximab, although the results of studies have been variable [3,15]. The prognostic value of genetic variants in ulcerative colitis (UC) seems to have fewer contradicting results, namely the association of HLA DRB*0103 with extensive and severe disease [16,17].

Previous studies indicated a strong impact of the protein called deleted in malignant brain tumours 1 (DMBT1) in intestinal inflammation [18]. The DMBT1 glycoprotein is majorly expressed in the mucosal epithelial cells in respiratory and gastrointestinal tracts [19,20,21,22]. The level of DMBT1 gene expression is upregulated in colon biopsies from CD patients in comparison to controls, and the levels downregulate with therapy [18,23]. Furthermore, DMBT1 has been reported to modulate bacterial recognition in epithelial cells, as a target for the NOD2 receptor [23]. Scientific research has shown that genetic variants of DMBT1 seem to contribute to the pathogenesis of adult CD [18]. Several DMBT1 SNPs have been found to relate to CD susceptibility, with the most strongly CD-associated SNP being the non-coding rs2981804, followed by rs2981745 [24]. Both of these variants were also associated with UC, although rs2981745 presented stronger association [24]. To date, data concern adult cohorts and have not included patients’ perspectives, such as whether specific DMBT1 polymorphisms are associated with more days spent in the hospital.

DMBT1 interacts with a number of different protein ligands, with the most studied interaction being the calcium-dependent interaction with surfactant protein D (SP-D). Surfactant protein D is encoded by the SFTPD gene and has immunomodulatory properties, while it also plays a role in regulating adaptive and innate immunity against commensal bacteria on mucosal surfaces, including the colon [21,25]. The rs2243639 polymorphism (Ala160Thr) in SFTPD has been shown to affect the oligomeric state of the molecule, the function of the protein, and the level of the protein in the blood [26]. Previous studies suggested that the rs2243639 polymorphism is significantly associated with susceptibility to UC in Japanese adults [27]. Another study presented contradicting results, showing a significant association of rs2243639 with susceptibility to CD, but not UC in Central Pennsylvania [28].

In the present study, we wished to evaluate the potential roles of the DMBT1 and SFTPD polymorphisms in the paediatric IBD cohort based on clinically relevant measures such as repeated flare-ups, the location of structural damage, number of hospitalisations, surgery, treatment, and overall patient’s medical history.

## 2. Materials and Methods

### 2.1. Patients

Patients recruited for the study were part of the POCOCO cohort (Polish Pediatric Crohn’s and Colitis cohort); specifically, there were 406 IBD patients (CD *n* = 214 and UC *n* = 192; F = 173, M = 233) (Table 1 and Table 2) recruited from The Department of Pediatric Gastroenterology and Metabolic Diseases, Poznań University of Medical Sciences; The Department of Gastroenterology, Hepatology, Feeding Disorders and Paediatrics, The Children’s Memorial Health Institute, Warsaw; The Department of Pediatric Gastroenterology and Nutrition, Medical University of Warsaw; The Department and Clinic of Pediatrics, Gastroenterology and Nutrition, Wroclaw Medical University; The Department of Pediatrics, Faculty of Medical Sciences in Zabrze, Medical University of Silesia, Katowice; The Department of Pediatrics, Faculty of Medical Sciences, Medical University of Silesia in Katowice; and The Department of Pediatric Endoscopy and Gastrointestinal Function Testing, Collegium Medicum in Bydgoszcz, Nicolaus Copernicus University in Toruń, Bydgoszcz. The inclusion criteria were age 3–18 and diagnosis of CD or UC. Patients in life-threatening, severe general condition, severe pain and distress, severe anaemia, or awaiting/having had urgent surgery were excluded from the study. The study was approved by the Bioethical Committee from the Poznan University of Medical Sciences (960/15 with the associated amendments).

### 2.2. Genotyping

The blood samples were collected into K2EDTA tubes and frozen in −20 °C. The DNA was extracted from whole blood by minicolumn purification method using the commercially available kit Blood Mini (A&A Biotechnology, Gdańsk, Poland). DNA from 1 mL of blood was eluted in 100 μL of Tris buffer (10 mM, pH 8.5) [29]. The genotyping was performed with the use of hydrolysis probe assay (TaqMan assay) with the following probes: C__26726205_30; C____347798_10; C___2804156_10 (Life Technologies Corp. Carlsbad, CA, USA). The reactions were performed on the CFX-96 thermocycler system (Bio-Rad, Hercules, CA, USA), with allele discrimination plots automatically constructed by CFX Manager software (Bio-Rad). The PCR cycling conditions were 60 °C for 30 s (pre-read), followed by 5 min at 95 °C (initial denature/enzyme activation), 40 cycles of 95 °C for 15 s (denature), 60 °C for 60 s (anneal/extend) and finally one cycle of 60 °C for 30 s (post-read).

### 2.3. Clinical Phenotype

Phenotypic data with demographic and clinical parameters were retrieved when available from medical files (hospital discharge and outpatient documents), blind to the results of the genotypic data. Due to a lack of consensus criteria defining disease severity over time, a customised questionnaire was created by experienced gastroenterologists including several aspects measured at diagnosis and at the time of the worst flare. The worst flare was defined by the highest Pediatric Crohn’s Disease Activity Index (PCDAI) and Pediatric Ulcerative Colitis Activity Index (PUCAI) in the medical history. The concentration of C-reactive protein (CRP) was collected by the time of diagnosis and the worst flare (CRP reference range 0–5 mg/L). Nutritional status included albumin concentration, body weight, height and body mass index (BMI) standardised to the reference Polish population [30]. The disease localisation was assessed with the Paris classification at the diagnosis and also at the worst flare [31]. The pharmacotherapy domain of the questionnaire included data on systemic steroid intake (number of separate courses), immunosuppressive and biologic treatment with time and age of the first intake, and operative treatment with time and age of the first intervention.

### 2.4. Disease Severity

The number of hospitalisations due to relapse and the total number of days spent in a hospital were calculated per 1 year of the disease from the patient’s clinical history. The number of relapses and severe relapses was collected and calculated per 1 year of the disease. The analysis also involved the presence of associated extraintestinal symptoms and other comorbidities.

### 2.5. Statistical Analysis

The normality of data distribution was tested with Shapiro–Wilk test. Medians and interquartile ranges were presented. All tests were considered statistically significant when *p* values < 0.05. Data were evaluated using the Statistica 13.1 software (StatSoft Inc., Tulsa, OK, USA), JASP 0.10.2 (University of Amsterdam, Amsterdam, The Netherlands) and Python programming language (Python Software Foundation). A two-tailed Fisher’s exact test was used for the analysis of categorical variables. Both models of inheritance (recessive and dominant) were assumed. The differences between genotypes were analysed using the Kruskal–Wallis test with Dunn’s post hoc comparisons. The differences within alleles were tested with the Mann–Whitney U test. The sample size was calculated prior to recruitment. Given the differences in relationships between polymorphisms, the severity of IBD would be 20% (SD = 50%, allocation factor = 1.5), and hence the sample size was 208 people (α = 0.05; β = 0 2).

## 3. Results

The nutritional status and clinical expression of UC and CD patients differed significantly (Supplementary Tables S1 and S2). Patients with CD had, most prevalently, ileocolonic localisation of the disease, both at the time of diagnosis and at the worst flare (Supplementary Table S3). Most frequently, the disease was non-stricturing, with no growth delay (Supplementary Table S3). UC patients had most frequently pancolitis (E4) and never severe behaviour because of their disease (S0) (Supplementary Table S4). The most common concomitant diseases reported in clinical history were allergy, celiac disease, bronchial asthma, obesity, gastroesophageal reflux disease, epilepsy and hypothyroidism. The most common extraintestinal manifestations were osteoporosis, arthritis, primary sclerosing cholangitis, arthralgia, osteopenia and erythema nodosum.

### 3.1. Genotyping

The genotyping was performed for all subjects studied (n = 406) (Table 1). No differences were observed in frequencies of any of the genotypes between CD/UC. The genotype frequencies of DMBT1 rs2981745 and SFTPD were in Hardy–Weinberg equilibrium (*p* = 0.102 and *p* = 0.629), whereas DMBT1 rs2981804 was in Hardy–Weinberg disequilibrium (*p* = 0.047).

The analysis of the linkage disequilibrium showed that the rs2981745(C) allele was correlated with the rs2981804(A) allele and the rs2981745(T) allele was correlated with the rs2981804(G) allele (D’: 1.0, r2: 0.5325 *p* = <0.001), whereas rs2981745 with rs2243639 and rs2981804 with rs2243639 were in linkage equilibrium (https://ldlink.nci.nih.gov/ accessed on 10 August 2020).

### 3.2. Association with Disease Severity

Patient’s age at diagnosis and at the worst flare did not differ between studied genotypes and alleles, nor did it differ for the whole group or within UC and CD groups. Patients with DMBT1 rs2981745 and TT genotype had more frequently penetrating and structuring (B2B3) behaviour of CD at diagnosis (TT: 9.1% vs. CT: 1.2% vs. CC: 1.0%; *p* = 0.032; Supplementary Table S5). Patients with UC and AA genotype of DMBT1 rs2981804 most frequently had severe behaviour (S1) of the disease (AA: 31.0% vs. AG: 19.0% vs. GG: 8.8%; *p* = 0.033; Supplementary Table S6). The nutritional status (body weight, body height, BMI with standardised values [z scores]) at diagnosis and at the time of worst flare did not differ between genotypes for IBD patients. However, patients with CD and the alternative allele (C+) of SFTPD had seemingly lower values of standardised body height at worst flare (CC/CT: −0.46 ([−1.34]−0.22) vs. TT: 0.10 ([−0.81]−0.66); *p* = 0.048). Patients with UC and reference allele (C+) of DMBT1 rs2981745 had higher values of standardised BMI at diagnosis (CC/CT: −0.52 ([−0.99]−0.16) vs. TT: −0.25 ([−1.31]−0.21]); *p* = 0.037) and at worst flare (CC/CT: −0.68 ([−1.12]−0.16) vs. TT: −0.52 ([−1.16]−0.07); *p* = 0.042).

#### 3.2.1. DMBT1 rs2981804

The albumin levels at diagnosis of IBD differed between genotypes (*p* = 0.022) with the highest values for carriers of AG genotype (Supplementary Table S7). The effect persisted for UC patients (*p* = 0.009), but not for CD (*p* = 0.271) (Table 2 and Table 3). The AA variant of DMBT1 rs2981804 was associated with the event of perforation in the course of the disease in CD patients (AA: 4.3% vs. AG/GG: 0.0%; *p* = 0.035). The frequency of biological treatment in IBD was higher among carriers of the risk genotype (AA) (AA: 48% vs. AG: 34% vs. GG = 34%; *p* = 0.034) (Supplementary Table S7), and this seemed to be true for UC patients (AA: 36% vs. AG: 23% vs. GG: 15%; *p* = 0.052) (Table 2), but not for CD (*p* = 0.338) (Table 3). Patients with IBD and the G+ allele had fewer biological agents than patients with AA (two and three agents AA: 7.0% vs. GG/AG: 5.8%, *p* = 0.018). This pertained only to UC patients (two or three agents AA: 8.6% vs. GG/AG: 4.5%; *p* = 0.025).

In the recessive model, patients with IBD and the AA variant had biological treatment more frequently than patients with AG/GG (AA: 48% vs. AG/GG: 34%; *p* = 0.012; OR = 0.57; 95%CI = 0.37–0.87). This was also true for infliximab alone (AA: 44 vs. AG/GG: 33%; *p* = 0.034; OR = 1.61 95%CI = 1.05–2.49). Only two patients took golimumab (all were carriers of the AA variant) and three patients took vedolizumab (two of them carrying the AA variant). In the dominant model, CD patients with the A+ allele had been treated with biological therapy earlier in the course of disease than carriers of the GG variant (AA/AG: 12.1 months (4.7–25.9) vs. GG: 25.1 (12.0–44.0); *p* = 0.031).

IBD patients with the risk genotype (AA) had, more frequently, other concomitant diseases (AA: 41% vs. AG: 33% vs. GG: 21%; *p* = 0.015) (Supplementary Table S7). This difference was observed only for CD patients (AA: 40% vs. AG: 29% vs. GG: 17%; *p* = 0.031) (Table 3). Similar, in the recessive model, patients with IBD and the AA variant also had, more frequently, other concomitant diseases (AA: 42% vs. AG/GG:31%; *p* = 0.034; OR = 1.60; 95% CI = (1.0.3–2.47). This association was observed only in the CD group (AA: 40% vs. AG/GG: 25%; *p* = 0.027; OR = 0.50; 95%CI = 0.27–0.92). Patients with CD and the AA variant had more cutaneous manifestations (AA: 19% vs. AG/GG: 6%; *p* = 0.008; OR = 0.29; 95%CI = 0.12–0.72).

#### 3.2.2. DMBT1 rs2981745

Patients with IBD and the TT genotype of DMBT1 rs2981745 had fewer events of severe relapses per 1 year of disease than the carriers of the rest of the genotypes (CC: 0.2 (0.0–0.5) vs. CT: 0.1 (0.0–0.5) vs. TT: 0.0 (0.0–0.3); *p* = 0.035) (Supplementary Table S8). This difference was true only for CD patients (CC: 0.3 (0.0–0.6) vs. CT: 0.2 (0.0–0.4) vs. TT: 0.0 (0.0–0.2); *p* = 0.020) (Table 4). Moreover, in the dominant model, CD patients with TT/CT had fewer severe relapses than patients with CC (TT/CT: 0.00 (0.00–0.38 vs. CC: 0.28 (0.00–0.54); *p* = 0.017). In the recessive model, patients with CD and the TT genotype had fewer severe relapses than carriers of CC/CT (TT: 0.00 (0.00–0.21) vs. CC/CT: 0.22 (0.00–0.49); *p* = 0.012).

In the group of UC, patients carrying the risk genotype (TT) had longer time to first dose of immunosuppression (TT: 20.5 months (8.0–32.4) vs. CT: 1.5 months (0.0–6.1) vs. CC: 4.1 months (1.0–10.0); *p* = 0.045) (Table 5). Additionally, in the recessive model, patients with UC and the TT variant took immunosuppressive treatment later in the disease course (TT: 10.16 (7.01–30.89) vs. CC/CT: 2.65 (0.00–9.61); *p* = 0.026). Patients with CD and the T+ allele had longer time to the first biological treatment (TT/CT 20.7 (8.5–37.4) months vs. CC: 11.0 (4.0–21.0); *p* = 0.015).

#### 3.2.3. SFTPD rs2243639

In IBD patients, the median age of first immunosuppression differed between SFTPD rs2243639 genotypes (CC: 12.6 (10.2–15.5) vs. CT: 12.3 (8.7–13.8) vs. TT: 13.4 (10.4–15.2); *p* = 0.048) (Supplementary Table S9). Patients with UC differed in number of steroid courses depending on SFTPD genotype (CC: 1 (0–2) vs. CT: 1 (1–3) vs. TT: 1 (1–3); *p* = 0.037) (Table 6). Patients with UC and the reference allele (T+) had, more frequently, systemic steroids than other patients (TT/CT: 77.0% vs. CC: 62.1%; *p* = 0.042), and significantly more courses of systemic steroids than the remaining patients (TT/CT: 1.0 (1.0–2.5) vs. CC: 1.0 (0.0–2.0); *p* = 0.013). Simultaneously, patients with UC and the T+ allele had less methotrexate treatment than other patients (TT/CT: 0.8% vs. CC: 7.6%; *p* = 0.019). No differences were observed in patients with CD (Supplementary Table S10).

## 4. Discussion

This study attempts to link the most potential IBD-associated SNPs in DMBT1 and SFTPD with the disease severity in children. We found that DMBT1 rs2981804 genotypes might be associated with albumin concentrations at diagnosis (IBD, UC), event of perforation (CD), frequency, number of agents and time to first biological treatment (IBD, UC, CD) (including infliximab (IBD)), frequency of cutaneous manifestations (CD) and the presence of concomitant diseases (IBD, CD) and severe behaviour of the UC disease. Furthermore, rs2981745 was significantly associated with the number of severe relapses per year of disease (IBD, CD) and time-to-biological treatment (CD). DMBT1 rs2981745 was also associated with penetrating and structuring (B2B3) behaviour of CD. In UC, rs2981745 genotypes might also be linked to time-to-immunosuppression, whereas SFTPD was seemingly found to be associated with age at first immunosuppression in IBD. We also found differences in the frequency of methotrexate and systemic steroid treatment and the total number of steroid courses (UC) depending on SFTPD genotype. CD patients with the alternative allele (C+) of SFTPD had lower standardised height at the worst flare, whereas patients with UC and reference allele (C+) of DMBT1 rs2981745 had higher standardised BMI at diagnosis and the worst flare.

Being in the non-coding region DMBT1, rs2981804 does not alter DMBT1 protein structure or function. However, previous data have shown that it is located within recognition sequences of transcription factors such as the cAMP-responsive element binding protein 1 (CREB1) and the activating transcription factor 2 (ATF-2) [24], and this could potentially affect the promotor activity of the protein and thereby the expression level of the protein. Differentially binding to DNA probes containing either the risk allele A or the protective G allele of SNP rs2981804 has shown that CREB1 and ATF-2 are involved in the transcriptional regulation of DMBT1 expression induced by Il-22 [24]. CREB1 mediates the transcription of genes containing the cAMP-responsive element, which involves genes coding interleukins IL-2, IL-6, IL-10 and TNF-α [32,33,34]. Phosphorylated CREB inhibits NF-κB activation and thereby limits proinflammatory responses [35]. CREB is also involved in the anti-apoptotic survival of macrophages and monocytes, as well as regulation of Th1, Th2, and Th17 responses and maintenance of regulatory T cells [36,37,38]. The other transcription factor, ATF-2, is involved in the transcription of inflammatory implicated genes such as proinflammatory cytokines, chemokines and cell adhesion molecules [39,40]. In animal studies, ATF-2 knockout inhibits TNF-α expression, while interleukin IL1β and IL-6 were also dramatically suppressed in ATF-2-deficient mice [40].

In the study of Diegelmann et al., the IBD risk allele of rs2981804 was found to be associated with lower DMBT1 gene expression in colonic tissue from CD patients. This association was independent of the NOD2 genotype, even though DMBT1 is a target for the intracellular pathogen receptor NOD2, known as the major CD susceptibility gene [24]. The authors suggested that this decreased expression in the colon contributes to increased CD susceptibility. However, this region might influence the expression of other genes, not solely DMBT1. Diegelmann et al. showed no significant relationships between rs2981804 and age at diagnosis, disease location and behaviour (Montreal classification), use of immunosuppressive agents, surgery and fistulas in adults with CD [24]. In our study, we showed a significant association of rs2981804 genotypes with perforation, other concomitant diseases and time-to-biological treatment.

For rs2981745 association with CD characteristics in adults, Diegelmann et al. showed significant relationships between the T allele and age at diagnosis ≤16 years, ileocolonic localisation (L3) and non-stricturing/non-penetrating behaviour of the disease (B1) [24]. Our study did not confirm the same results, but we found that rs2981745 in CD might be linked to the number of severe relapses. In UC adults, Diegelmann et al. showed differences in age of diagnosis and frequency of left-sided (E2) and extensive UC (E3) [24]. Our study does not repeat those findings. However, we found an association with the time-to-immunosuppression and standardised BMI at diagnosis and the worst flare.

The DMBT1 protein has been implicated in IBD for some time now. It has been found that DMBT1 mRNA expression levels are elevated in tissues of adults with IBD (20 IBD biopsies and nine healthy controls) and correlate with both the Crohn’s Disease Endoscopic Index of Severity (CDEIS) for CD and the clinical activity index (CAI) for UC [18]. Furthermore, the levels of expression increased with disease activity. In our study, we did not find any significant associations between DMBT1 polymorphisms and disease activity scales in children. Another study confirmed DMBT1 upregulation in colon biopsies from CD patients compared with controls, and also demonstrated its downregulation with anti-IL23 therapy [23].

Currently, there are no data on SFTPD rs2243639 associations with disease severity in IBD. A study of 256 IBD adults vs. 374 healthy subjects showed rs2243639 susceptibility to CD (*p* = 0.004), but not UC (*p* = 0.883) [28]. Our findings suggested that the presence of the T allele might be associated with the frequency and number of steroid courses in children with UC. The SFTPD gene might also be linked with age at first immunosuppression in IBD patients. In children with CD, we found a significant association with standardised height at the worst flare.

To date, gastroenterologists have lacked the markers that could predict the unforeseeable course of disease, indicating which patients would be in need of intensive treatment [41], while the available tools are also imperfect [42]. Previous case–control studies have already shown the allele frequencies of DMBT1 and SFTPD in IBD adults compared to controls [24,27,28], whereas our study focuses on extensive associations of the selected variants with disease severity in children. In our paediatric cohort, we used severity measures such as number of hospitalisations and days spent in hospital to add on to the field of antimicrobial peptides biology with the patient-orientated perspective. However, this study has some limitations; for example, it is still challenging to define global severity in the IBD course as the condition is complex. Longer follow-up regarding the need for surgery would also help to describe the severity. Additionally, the clinical data from diagnosis and the worst flare were collected retrospectively.

## 5. Conclusions

In the present study, we found that selected polymorphisms of DMBT1 and SFTPD might be associated with some disease severity measures in children with IBD. However, the magnitude of associations and their clinical relevance might be minor.

## Figures and Tables

**Table 1:** DMBT1 (rs2981804, rs2981745) and SFTPD (rs2243639) genotype distribution.

	UC *n* = 192	CD *n* = 214	*p* Value *	OR (95% CI)
rs2981804				
AA	58 (30.2)	70 (32.7)	0.595	0.89 (0.59–1.36)
AG	100 (52.1)	98 (45.8)	0.233	1.29 (0.87–1.90)
GG	34 (17.7)	46 (21.5)	0.382	0.79 (0.48–1.29)
rs2981745				
TT	14 (7.3)	22 (10.3)	0.342	0.69 (0.34–1.39)
CT	87 (45.3)	87 (40.7)	0.366	1.22 (0.82–1.81)
CC	91 (47.4)	105 (49.0)	0.766	0.94 (0.63–1.38)
rs2243639				
TT	28 (14.6)	34 (15.9)	0.783	0.90 (0.53–1.56)
CT	98 (51.0)	105 (49.1)	0.766	1.08 (0.73–1.60)
CC	66 (34.4)	75 (35.0)	0.917	0.97 (0.65–1.46)

* Two-tailed Fisher’s exact test.

**Table 2:** Association between DMBT1 rs2981804 genotypes and UC characteristics.

Variables Median (IQR) or *n* (%)	AA	AG	GG	*p* Value *
**Selected biochemical parameters**				
CRP at diagnosis (mg/L)	2.5 (0.6–9.3)	1.7 (0.5–9.6)	5.6 (0.7–16.2)	0.173
CRP at worst flare (mg/L)	2.7 (0.6–14.2)	2.4 (0.5–11.2)	3.9 (1.0–27.8)	0.445
Albumin level at diagnosis (g/dL)	4.0 (3.5–4.3)	4.2 (4.0–4.5)	4.0 (3.4–4.4)	**0.009** ^1^
Albumin level at worst flare (g/dL)	4.0 (3.7–4.4)	4.2 (3.9–4.5)	4.3 (3.8–4.4)	0.307
**Disease activity scales**				
PUCAI at diagnosis	50 (40–65)	45 (25–60)	41 (28–53)	0.125
PUCAI at worst flare	55 (40–65)	50 (33–65)	53 (35–65)	0.707
**Treatment**				
Systemic steroids	45 (78)	72 (72)	21 (62)	0.265
Number of courses of steroid treatment	1 (1–2)	1 (1–2)	1 (0–2)	0.631
Immunosuppressive treatment	36 (63)	60 (60)	16 (47)	0.296
Number of immunosuppressants	1 (0–1)	1 (0–1)	0 (0–1)	0.449
Time-to-first dose of immunosuppressive treatment (months)	4.0 (1.0–10.0)	3.0 (0.3–10.0)	1.9 (0.0–10.1)	0.682
Age at first intake of immunosuppressive treatment (years)	10.2 (6.7–14.0)	11.6 (7.8–14.7)	13.2 (8.9–14.7)	0.460
Biological therapy	21 (36)	23 (23)	5 (15)	0.052
Time-to-first dose of biological treatment (months)	18.4 (10.0–28.1)	12.5 (7.2–25.1)	12.2 (4.0–29.0)	0.570
Age at first biological treatment	11.7 (6.6–15.3)	13.1 (7.9–15.6)	10.7 (5.3–11.8)	0.271
Operative treatment	1 (2)	3 (3)	0 (0)	0.557
Age at first surgery (years)	15.6	10.0 (6.8–14.8)	-	1.000
Time-to-first surgery (months)	0.9	22.7 (10.9–33.0)	-	1.000
Hospitalisations for relapse (per 1 year of the disease)	0.6 (0.3–0.9)	0.6 (0.3–1.3)	1.0 (0.4–1.6)	0.335
Days of hospitalisation for relapse (per 1 year of the disease)	4.0 (1.5–7.1)	5.0 (1.3–9.7)	5.4 (2.5–10.3)	0.721
Relapses from diagnosis (per 1 year of the disease)	0.5 (0.3–0.7)	0.6 (0.3–1.3)	1.0 (0.3–1.8)	0.378
Severe relapses from diagnosis (per 1 year of the disease)	0.1 (0.0–0.4)	0.0 (0.0–0.6)	0.3 (0.0–0.4)	0.900
**Concomitant diseases**	24 (41)	37 (37)	9 (26)	0.353
**Extraintestinal manifestations**	11 (19)	23 (23)	3 (9)	0.194

* Kruskal–Wallis test. Bold means *p* < 0.05. ^1^ Dunn’s post hoc comparison: AA vs. AG *p* = 0.005 (Bonferroni and Holm).

**Table 3:** Association between DMBT1 rs2981804 genotypes and CD characteristics.

Variables Median (IQR) or *n* (%)	AA	AG	GG	*p* Value *
**Selected biochemical parameters**				
CRP at diagnosis (mg/L)	14.4 (2.4–37.8)	13.2 (2.1–25.0)	9.4 (1.9–24.9)	0.395
CRP at worst flare (mg/L)	14.7 (3.0–34.0)	12.7 (2.6–30.0)	14.2 (3.1–39.1)	0.829
Albumin level at diagnosis (g/dL)	3.8 (3.4–4.2)	3.9 (3.5–4.3)	4.0 (3.7–4.5)	0.271
Albumin level at worst flare (g/dL)	3.9 (3.5–4.2)	4.0 (3.5–4.3)	3.8 (3.6–4.1)	0.638
**Disease activity scales**				
PCDAI at diagnosis	30 (23–50)	35 (23–50)	30 (23–43)	0.677
PCDAI at worst flare	41 (30–53)	40 (25–53)	48 (30–53)	0.629
**Treatment**				
Systemic steroids	35 (50)	52 (53)	28 (61)	0.509
Number of courses of steroid treatment	1 (0–2)	1 (0–1)	1 (1–2)	0.523
Immunosuppressive treatment	55 (79)	76 (78)	37 (80)	0.926
Number of immunosuppressants	1 (1–1)	1 (1–1)	1 (1–1)	0.636
Time-to-first dose of immunosuppressive treatment (months)	2.5 (0.0–12.0)	1.0 (0.0–6.8)	0.7 (0.0–15.0)	0.377
Age at first intake of immunosuppressive treatment (years)	13.1 (11.3–14.9)	12.2 (9.4–14.9)	13.0 (9.2–13.9)	0.135
Biological therapy	40 (57)	45 (46)	22 (48)	0.338
Number of biological agents	1 (0–1)	0 (0–1)	0 (0–1)	0.538
Time-to-first dose of biological treatment (months)	11.4 (4.0–21.0)	13.0 (6.0–28.3)	25.1 (12.0–44.0)	0.074
Age at first biological treatment	13.5 (12.5–14.8)	13.9 (10.8–15.4)	14.1 (11.3–15.1)	0.969
Operative treatment	12 (17)	12 (12)	5 (11)	0.558
Age at first surgery (years)	14.5 (13.1–16.3)	13.8 (11.7–15.6)	14.9 (14.5–15.7)	0.675
Time-to-first surgery (months)	8.0 (0.0–41.1)	35.0 (23.1–56.8)	11.5 (3.0–27.0)	0.259
**Hospitalisations (if duration ≥1 years)**				
Hospitalisations for relapse (per 1 year of the disease)	0.4 (0.3–0.9)	0.5 (0.2–0.9)	0.5 (0.3–0.9)	0.925
Days of hospitalisation for relapse (per 1 year of the disease)	3.7 (1.6–8.5)	4.5 (1.2–7.2)	3.5 (1.0–6.5)	0.949
Relapses from diagnosis (per 1 year of the disease)	0.5 (0.3–0.9)	0.5 (0.2–0.9)	0.4 (0.2–1.1)	0.916
Severe relapses from diagnosis (per 1 year of the disease)	0.3 (0.0–0.5)	0.2 (0.0–0.5)	0.0 (0.0–0.3)	0.116
**Concomitant diseases**	28 (40)	28 (29)	8 (17)	**0.031** ^1^
**Extraintestinal manifestations**	18 (26)	28 (29)	7 (15)	0.218

* Kruskal–Wallis test. Bold means *p* < 0.05. ^1^ Dunn’s post hoc comparison: AA vs. GG *p* = 0.014 (Bonferroni and Holm).

**Table 4:** Association between DMBT1 rs2981745 genotypes and CD characteristics.

Variables Median (IQR) or *n* (%)	CC	CT	TT	*p* Value *
**Selected biochemical parameters**				
CRP at diagnosis (mg/L)	13.6 (2.6–31.1)	13.0 (2.0–28.1)	9.8 (2.0–18.3)	0.518
CRP at worst flare (mg/L)	11.9 (2.1–32.0)	14.2 (3.2–39.4)	14.7 (7.1–30.2)	0.776
Albumin level at diagnosis (g/dL)	3.8 (3.5–4.2)	3.9 (3.5–4.3)	4.0 (3.7–4.4)	0.675
Albumin level at worst flare (g/dL)	3.9 (3.5–4.3)	3.9 (3.5–4.2)	3.9 (3.5–4.2)	0.949
**Disease activity scales**				
PCDAI at diagnosis	30 (23–48)	35 (25–50)	25 (25–39)	0.262
PCDAI at worst flare	40 (30–53)	43 (30–53)	35 (28–53)	0.801
**Treatment**				
Systemic steroids	55 (52)	45 (52)	15 (68)	0.358
Number of courses of steroid treatment	1 (0–2)	1 (0–2)	1 (1–2)	0.875
Immunosuppressive treatment	81 (77)	69 (79)	18 (82)	0.865
Number of immunosuppressants	1 (1–1)	1 (1–1)	1 (1–1)	0.634
Time-to-first dose of immunosuppressive treatment (months)	1.6 (0.0–7.0)	2.0 (0.0–7.8)	0.5 (0.0–17.3)	0.981
Age at first intake of immunosuppressive treatment (years)	13.1 (10.7–14.7)	12.3 (9.2–14.9)	13.0 (9.7–14.2)	0.315
Biological therapy	57 (54)	38 (44)	12 (55)	0.311
Number of biological agents	1 (0–1)	0 (0–1)	1 (0–1)	0.379
Time-to-first dose of biological treatment (months)	11.0 (4.1–20.8)	20.4 (9.6–35.0)	20.3 (7.6–40.2)	0.055
Age at first biological treatment	13.6 (12.5–15.1)	13.8 (10.9–15.5)	14.2 (10.8–14.8)	0.810
Operative treatment	18 (17)	7 (8)	4 (18)	0.150
Age at first surgery (years)	14.0 (11.6–15.6)	15.2 (14.0–15.7)	14.7 (14.2–15.3)	0.475
Time-to-first surgery (months)	10.0 (2.2–41.6)	29.1 (27.0–56.8)	7.3 (2.3–15.5)	0.154
**Hospitalisations (if duration ≥1 years)**				
Hospitalisations for relapse (per 1 year of the disease)	0.5 (0.3–0.9)	0.5 (0.2–0.7)	0.5 (0.3–0.9)	0.588
Days of hospitalisation for relapse (per 1 year of the disease)	4.6 (1.8–7.6)	3.5 (1.2–7.1)	2.7 (0.9–6.5)	0.504
Relapses from diagnosis (per 1 year of the disease)	0.6 (0.3–0.9)	0.4 (0.2–0.8)	0.4 (0.0–1.1)	0.208
Severe relapses from diagnosis (per 1 year of the disease)	0.3 (0.0–0.6)	0.2 (0.0–0.4)	0.0 (0.0–0.2)	**0.020** ^1^
**Concomitant diseases**	36 (34)	24 (28)	4 (18)	0.269
**Extraintestinal manifestations**	30 (29)	20 (23)	3 (14)	0.297

* Kruskal–Wallis test. Bold means *p* < 0.05. ^1^ Dunn’s post hoc comparison: CC vs. TT *p* = 0.011 (Bonferroni and Holm).

**Table 5:** Association between DMBT1 rs2981745 genotypes and UC characteristics.

Variables Median (IQR) or *n* (%)	CC	CT	TT	*p* Value *
**Selected biochemical parameters**				
CRP at diagnosis (mg/L)	2.2 (0.6–10.2)	1.9 (0.4–7.8)	9.2 (3.7–21.5)	0.057
CRP at worst flare (mg/L)	2.6 (0.6–13.3)	2.2 (0.5–7.1)	7.9 (3.6–28.4)	0.120
Albumin level at diagnosis (g/dL)	4.1 (3.6–4.4)	4.2 (3.8–4.5)	4.0 (3.5–4.4)	0.504
Albumin level at worst flare (g/dL)	4.1 (3.7–4.4)	4.2 (3.9–4.5)	4.2 (3.8–4.4)	0.309
**Disease activity scales**				
PUCAI at diagnosis	50 (30–60)	45 (25–60)	40 (33–50)	0.373
PUCAI at worst flare	55 (36–65)	50 (35–65)	50 (43–65)	0.956
**Treatment**				
Systemic steroids	71 (78)	60 (69)	7 (50)	0.069
Number of courses of steroid treatment	1 (1–2)	1 (0–2)	1 (0–2)	0.250
Immunosuppressive treatment	56 (62)	52 (60)	4 (29)	0.058
Number of immunosuppressants	1 (0–1)	1 (0–1)	0 (0–1)	0.084
Time-to-first dose of immunosuppressive treatment (months)	4.1 (1.0–10.0)	1.5 (0.0–6.1)	20.5 (8.0–32.4)	**0.045** ^1^
Age at first intake of immunosuppressive treatment (years)	11.5 (6.8–14.4)	11.9 (9.2–14.6)	13.7 (12.0–14.6)	0.632
Biological therapy	28 (31)	20 (23)	1 (7)	0.130
Time-to-first dose of biological treatment (months)	17.0 (10.0–26.9)	12.0 (5.6–23.5)	29.0	0.247
Age at first biological treatment	13.1 (6.6–15.3)	11.9 (10.0–14.7)	10.7	0.632
Operative treatment	2 (2)	2 (2)	0 (0)	0.851
Age at first surgery (years)	11.2 (9.0–13.4)	10.4 (9.6–14.8)		1.000
Time-to-first surgery (months)	8.8 (4.8–12.7)	28.7 (16.8–33.0)		0.248
**Hospitalisations (if duration ≥1 years)**				
Hospitalisations for relapse (per 1 year of the disease)	0.6 (0.3–1.2)	0.7 (0.3–1.4)	0.7 (0.5–1.0)	0.972
Days of hospitalisation for relapse (per 1 year of the disease)	5.2 (1.6–9.5)	4.6 (1.7–9.0)	5.4 (3.1–6.5)	0.897
Relapses from diagnosis (per 1 year of the disease)	0.6 (0.3–1.1)	0.6 (0.3–1.3)	0.6 (0.3–1.4)	0.944
Severe relapses from diagnosis (per 1 year of the disease)	0.1 (0.0–0.4)	0.0 (0.0–0.6)	0.0 (0.0–0.2)	0.709
**Concomitant diseases**	38 (42)	29 (33)	3 (21)	0.242
**Extraintestinal manifestations**	18 (20)	18 (21)	1 (7)	0.484

* Kruskal–Wallis test. Bold means *p* < 0.05. ^1^ Dunn’s post hoc comparison: CT vs. TT *p* = 0.055 (Bonferroni and Holm).

**Table 6:** Association between SFTPD rs2243639 genotypes and UC characteristics.

Variables Median (IQR) or *n* (%)	CC	CT	TT	*p* Value *
**Selected biochemical parameters**				
CRP at diagnosis (mg/L)	2.2 (0.5–9.1)	2.1 (0.4–10.5)	2.9 (1.0–18.0)	0.672
CRP at worst flare (mg/L)	2.1 (0.6–7.5)	2.9 (0.6–14.7)	2.7 (1.0–19.0)	0.716
Albumin level at diagnosis (g/dL)	4.2 (3.8–4.5)	4.1 (3.5–4.4)	4.1 (3.8–4.3)	0.247
Albumin level at worst flare (g/dL)	4.2 (3.8–4.4)	4.1 (3.7–4.5)	4.1 (3.9–4.3)	0.594
**Disease activity scales**				
PUCAI at diagnosis	45 (30–55)	45 (25–58)	45 (28–68)	0.813
PUCAI at worst flare	50 (35–65)	53 (35–65)	60 (38–65)	0.846
**Treatment**				
Systemic steroids	41 (62)	77 (79)	20 (71)	0.072
Number of courses of steroid treatment	1 (0–2)	1 (1–3)	1 (1–3)	**0.037** ^1^
Immunosuppressive treatment	35 (53)	59 (61)	18 (64)	0.495
Number of immunosuppressants	1 (0–1)	1 (0–1)	1 (0–1)	0.706
Time-to-first dose of immunosuppressive treatment (months)	2.1 (0.0–8.4)	3.5 (0.1–10.1)	5.2 (1.8–11.0)	0.392
Age at first intake of immunosuppressive treatment (years)	13.0 (7.9–15.6)	11.3 (7.5–13.7)	12.8 (9.1–14.9)	0.334
Biological therapy	18 (27)	23 (23)	8 (29)	0.795
Number of biological agents	0 (0–1)	0 (0–0)	0 (0–1)	0.736
Time-to-first dose of biological treatment (months)	13.5 (4.5–20.8)	20.1 (10.2–29.1)	12.1 (6.0–23.3)	0.258
Age at first biological treatment	12.5 (8.7–15.3)	11.0 (6.9–14.5)	12.1 (7.1–15.6)	0.710
Operative treatment	3 (5)	0 (0)	1 (4)	0.115
Age at first surgery (years)	12.6 (9.8–15.4)		6.8	0.317
Time-to-first surgery (months)	16.8 (4.0–30.8)		16.7	1.000
**Hospitalisations (if duration ≥1 years)**				
Hospitalisations for relapse (per 1 year of the disease)	0.7 (0.2–1.4)	0.6 (0.3–1.1)	0.9 (0.4–1.5)	0.854
Days of hospitalisation for relapse (per 1 year of the disease)	3.6 (1.2–9.2)	5.2 (2.3–7.7)	7.7 (2.7–13.8)	0.492
Relapses from diagnosis (per 1 year of the disease)	0.6 (0.2–1.1)	0.6 (0.3–1.1)	0.9 (0.3–1.3)	0.925
Severe relapses from diagnosis (per 1 year of the disease)	0.1 (0.0–0.4)	0.1 (0.0–0.5)	0.1 (0.0–0.5)	0.889
**Concomitant diseases**	20 (30)	41 (42)	9 (32)	0.283
**Extraintestinal manifestations**	16 (24)	14 (14)	7 (25)	0.201

* Kruskal–Wallis test. Bold means *p* < 0.05. ^1^ Dunn’s post hoc comparison: CC vs. CT *p* = 0.016 (Bonferroni and Holm).

## Data Availability

The data are not publicly available due to privacy and ethical restrictions.

## Supplementary Materials

The following are available online at https://www.mdpi.com/article/10.3390/children8110946/s1, Table S1: Demographic characteristics of paediatric patients with inflammatory bowel disease, Table S2: The clinical expression of Crohn’s disease and ulcerative colitis, Table S3: Disease characteristics of the patients with Crohn’s disease enrolled in the study, Table S4: Disease characteristics of the patients with ulcerative colitis enrolled in the study, Table S5: Disease localisation and behaviour according to Paris Classification in patients with Crohn’s disease depending on DMBT1 rs2981745 genotype, Table S6: Disease localisation and behaviour according to Paris Classification in patients with ulcerative colitis depending on DMBT1 rs2981804 genotype, Table S7: Association between DMBT1 rs2981804 genotypes and IBD characteristics, Table S8: Association between DMBT1 rs2981745 genotypes and IBD characteristics, Table S9: Association between SFTPD rs2243639 genotypes and IBD characteristics, Table S10: Association between SFTPD rs2243639 genotypes and CD characteristics.
