# Supplementary material for: Genetic Variants of DMBT1 and SFTPD and Disease Severity in Paediatric Inflammatory Bowel Disease—A Polish Population-Based Study
Source: Children (Basel). 2021 Oct 21;8(11):946. doi: 10.3390/children8110946 (PMC8618964; doi:10.3390/children8110946)
Supplement: Supplementary file 1 [file children-08-00946-s001.zip › children-1401492-supplementary.pdf]

Table S1. Demographic characteristics of pediatric patients with inflammatory bowel disease.

Variables<br>Median (IQR) or n (%)	n	Crohn's disease	Ulcerative colitis	p value
Age [y]				
At inclusion	397	15.18 (13.32-17.05)	15.11 (11.70-16.75)	0.044
At diagnosis	404	12.58 (10.02-14.32)	12.14 (7.89-14.94)	0.365
At worst flare	355	13.63 (11.54-15.85)	13.76 (10.13-15.84)	0.244
Duration of the disease [y]	390	2.23 (0.82-4.25)	1.88 (0.36-3.77)	0.239
Body weight at diagnosis [kg]	387	38.0 (27.0-49.8)	40.0 (27.8-53.9)	0.490
Body weight at diagnosis [z score]	383	-0.82 ([-1.39]-[-0.04]	-0.51 ([-1.12]-0.22)	0.003
Body height at diagnosis [cm]	382	151.0 (137.0-164.5)	152.0 (130.5-168.3)	0.718
Body height at diagnosis [z score]	378	-0.37 ([-1.29]-0.47)	0.06 ([-0.67]-0.81]	0.001
Body mass index at diagnosis [kg/m^2]	382	16.6 (14.5-18.4)	17.4 (15.5-19.3)	0.019
Body mass index at diagnosis [z score]	378	-0.79 ([-1.47]-[-0.04])	-0.49 ([-1.00]-0.16)	0.006
Body weight at worst flare [kg]	345	42.7 (31.1-53.0)	46.9 (31.5-56.6)	0.449
Body weight at worst flare [z score]	339	-0.90 ([-1.43]-[-0.16])	-0.55 ([-1.00]-0.27)	<0.001
Body height at worst flare [cm]	345	158.0 (145.0-168.0)	159.5 (140.0-172.0)	0.791
Body height at worst flare [z score]	340	-0.42 ([-1.30]-0.30)	-0.02 ([-0.67]-0.80)	<0.001
Body mass index at worst flare [kg/m^2]	341	17.0 (15.1-19.5)	17.5 (15.8-20.3)	0.083
Body mass index at worst flare [z score]	337	-0.93 ([-1.46]-0.05)	-0.65 ([-1.12]-0.16	0.012

Table S2. The clinical expression of Crohn's disease and ulcerative colitis.

Variables<br>Median (IQR) or n (%)	Crohn's disease	Ulcerative colitis	p value
**Selected parameters of inflammation**			
CRP at diagnosis [mg/l]	12.94 (2.10-29.25)	2.24 (0.50-10.80)	<0.001
CRP at worst flare [mg/l]	13.95 (3.03-32.43)	2.70 (0.63-13.44)	<0.001
Albumin level at diagnosis [g/dl]	3.90 (3.51-4.25)	4.10 (3.70-4.40)	<0.003
Albumin level at worst flare [g/dl]	3.90 (3.50-4.20)	4.18 (3.80-4.41)	<0.001
**Disease activity scales**			
PCDAI/PUCAI at diagnosis	32 (23-48)	45 (28-60)	
PCDAI/PUCAI at worst flare	40 (30-53)	50 (35-65)	
**Treatment**			
Systemic steroids*	115 (53.7)	138 (71.9)	<0.001
Number of courses of steroid treatment	1 (0-2)	1 (1-2)	0.004
Immunosuppressive treatment**	168 (78.5)	112 (58.6)	<0.001
Number of immunosuppressants	1 (1-1)	1 (0-1)	0.002
Time-to-first dose of immunosuppressive treatment [months]	1.58 (0.00-8.45)	3.04 (0.00-10.00)	0.173
Age at first intake of immunosuppressive treatment [years]	12.82 (10.42-14.67)	11.63 (7.82-14.63)	0.049
Biological therapy***	107 (50.0)	49 (25.5)	<0.001
Total number of biologics	0 (0-1)	0 (0-1)	<0.001
Time-to-first dose of biological treatment [months]	14.05 (5.99-26.81)	14.05 (8.60-26.96)	0.650
Age at first biological treatment	13.62 (11.66-15.25)	11.93 (7.20-15.28)	0.025
Operative treatment****	29 (13.6)	4 (2.1)	<0.001
Age at first surgery [years]	14.45 (13.22-15.67)	10.41 (8.21-15.20)	0.270
Time-to-first surgery [months]	23.13 (3.03-41.09)	16.68 (5.03-28.65)	0.738
**Hospitalisations (if duration ≥1 years)**			
Hospitalisations for relapse (per 1 year of the disease)	0.49 (0.22-0.87)	0.65 (0.28-1.29)	0.020
Days of hospitalisation for relapse (per 1 year of the disease)	3.91 (1.33-7.24)	4.72 (1.77-8.92)	0.173
Relapses from diagnosis (per 1 year of the disease)	0.48 (0.20-0.87)	0.61 (0.30-1.19)	0.044
Severe relapses from diagnosis (per 1 year of the disease)	0.19 (0.00-0.47)	0.11 (0.00-0.49)	0.419
**Concomitant diseases*****	64 (29.9)	70 (36.5)	0.171
**Extraintestinal manifestations**	53 (24.8)	37 (19.3)	0.190

* Systemic steroid therapy included: methylprednisolone, prednisone, hydrocortisone.

** Immunosuppressive and anti-inflammatory agents included: azathioprine, methotrexate, mercaptopurine, cyclosporine, mycophenolate mofetil, tacrolimus, sulfasalazine.

*** Biological agents included: infliximab, adalimumab, golimumab, vedolizumab.

**** Only surgery related to IBD-specific problems (e. g. colectomy, resection, fistula, perforation, abscess) was included.

***** e.g. celiac disease, bronchial asthma, obesity, gastroesophageal reflux disease, epilepsy, hypothyroidism.

Table S3. Disease characteristics of the patients with Crohn's disease enrolled in the study.

Paris Classification		At diagnosis n (%)	At worst flare n (%)
Age	A1a: 0-10 years	53 (24.8)	21 (9.8)
	A1b: 10-17 years	148 (69.2)	142 (66.4)
	A2: 10-40 years	12 (5.6)	21 (9.8)
Location	L1: Ileal	52 (24.3)	40 (18.7)
	L2: Colonic	39 (18.3)	27 (12.6)
	L3: Ileocolonic	98 (45.8)	93 (43.7)
	L4a: upper disease proximal to ligament of Treitz	22 (10.3)	18 (8.4)
	L4b: upper disease distal to ligament of Treitz	8 (3.7)	9 (4.2)
Behaviour	B1: non-stricturing	148 (69.5)	116 (54.5)
	B2: structuring	15 (7.0)	19 (8.9)
	B3: penetrating	19 (8.9)	21 (9.8)
	B2B3: penetrating and stricturing	4 (1.9)	5 (2.3)
	P: perianal disease modifier	20 (9.3)	20 (9.3)
Growth	G0: no evidence of growth delay	147 (69.0)	124 (58.2)
	G1: growth delay	33 (15.4)	34 (15.9)

Table S4. Disease characteristics of the patients with ulcerative colitis enrolled in the study.

Paris Classification		At diagnosis n (%)	At worst flare n (%)
Extent	E1: Proctitis	19 (9.9)	9 (4.7)
	E2: Left-sided colitis	33 (17.2)	28 (14.6)
	E3: Extensive colitis	30 (15.6)	24 (12.5)
	E4: Pancolitis	90 (46.9)	82 (42.7)
Severity	S0: never severe	116 (60.4)	89 (46.4)
	S1: ever severe	40 (20.8)	52 (27.1)

Table S5. Disease localisation and behaviour according to Paris Classification in patients with Crohn's disease depending on *DMBT1* rs2981745 genotype.

Paris Classification n (%)		DMBT1 rs2981745							
		At diagnosis				At worst flare			
		CC	CT	TT	p	CC	CT	TT	p
Location	L1	26 (24.8)	19 (22.1)	7 (31.8)	0.6353	18 (17.1)	18 (20.9)	4 (18.2)	0.7992
	L2	19 (18.1)	15 (17.4)	5 (22.7)	0.8470	15 (14.3)	8 (9.3)	4 (18.2)	0.4222
	L3	51 (48.6)	40 (46.5)	7 (31.8)	0.3570	44 (41.9)	40 (46.5)	9 (40.9)	0.7861
	L4a	10 (9.5)	12 (14.0)	0 (0.0)	0.1489	7 (6.7)	11 (12.8)	0 (0.0)	0.1036
	L4b	4 (3.8)	4 (4.7)	0 (0.0)	0.5930	5 (4.8)	4 (4.7)	0 (0.0)	0.5831
Behaviour	B1	75 (71.4)	57 (66.3)	16 (72.7)	0.7013	56 (53.3)	47 (54.7)	13 (59.1)	0.8851
	B2	6 (5.7)	8 (9.3)	1 (4.5)	0.5605	8 (7.6)	7 (8.1)	4 (18.2)	0.2734
	B3	11 (10.5)	8 (9.3)	0 (0.0)	0.2906	11 (10.5)	10 (11.6)	0 (0.0)	0.2540
	B2B3	1 (1.0)	1 (1.2)	2 (9.1)	**0.0317**	3 (2.9)	1 (1.2)	1 (4.5)	0.5758
	p	10 (9.5)	7 (8.1)	3 (13.6)	0.7321	8 (7.6)	10 (11.6)	2 (9.1)	0.6404
Growth	G0	78 (74.3)	54 (62.8)	15 (68.2)	0.2328	61 (58.1)	51 (59.3)	12 (54.5)	0.9215
	G1	18 (17.1)	12 (14.0)	3 (13.6)	0.8066	18 (17.1)	10 (11.6)	6 (27.3)	0.1831

L1: Ileal; L2: Colonic; L3: Ileocolonic; L4a: upper disease proximal to ligament of Treitz; L4b: upper disease distal to ligament of Treitz; B1: non-stricturing; B2: structuring; B3: penetrating; B2B3: penetrating and structuring; p: perianal disease modifier; G0: no evidence of growth delay; G1: growth delay

Table S6. Disease localisation and behaviour according to Paris Classification in patients with ulcerative colitis depending on *DMBT1* rs2981804 genotype.

Paris Classification n (%)		DMBT1 rs2981804							
		At diagnosis				At worst flare			
		AA	AG	GG	p	AA	AG	GG	p
Extent	E1	6 (10.3)	9 (9.0)	4 (11.8)	0.8891	4 (6.9)	3 (3.0)	2 (5.9)	0.6035
	E2	8 (13.8)	20 (20.0)	5 (14.7)	0.5581	10 (17.2)	12 (12.0)	6 (17.6)	0.5750
	E3	10 (17.2)	14 (14.0)	6 (17.6)	0.8112	8 (13.8)	12 (12.0)	4 (11.8)	0.9382
	E4	29 (50.0)	47 (47.0)	14 (41.2)	0.7161	25 (43.1)	44 (44.0)	13 (38.2)	0.8403
Severity	S0	29 (50.0)	65 (65.0)	22 (64.7)	0.1532	24 (41.4)	50 (50.0)	15 (44.1)	0.5560
	S1	18 (31.0)	19 (19.0)	3 (8.8)	**0.0334**[1]	21 (36.2)	22 (22.0)	9 (26.5)	0.1541

E1: Proctitis; E2: Left-sided colitis; E3: Extensive colitis; E4: Pancolitis; S0: never severe; S1: ever severe

[1] post hoc comparison: AA vs. GG p=0.0173 (Bonferroni and Holm)

Table S7. Association between *DMBT1* rs2981804 genotypes and IBD characteristics.

Variables<br>Median (IQR) or n (%)	AA	AG	GG	p value
**Selected biochemical parameters**				
CRP at diagnosis [mg/l]	6.7 (1.3-22.9)	5.0 (0.8-19.2)	7.0 (1.1-22.1)	0.4166
CRP at worst flare [mg/l]	6.6 (1.3-28.3)	4.8 (1.0-21.1)	8.9 (1.9-33.3)	0.2304
Albumin level at diagnosis [g/dl]	3.9 (3.5-4.2)	4.1 (3.7-4.4)	4.0 (3.6-4.4)	**0.0222**[1]
Albumin level at worst flare [g/dl]	4.0 (3.5-4.3)	4.0 (3.6-4.4)	4.0 (3.6-4.3)	0.3115
**Treatment**				
Systemic steroids	80 (63)	124 (63)	49 (61)	0.9760
Number of courses of steroid treatment	1 (1-2)	1 (0-2)	1 (0-2)	0.7816
Immunosuppressive treatment	91 (72)	136 (69)	53 (66)	0.7018
Number of immunosuppressants	1 (0-1)	1 (0-1)	1 (0-1)	0.6879
Time-to-first dose of immunosuppressive treatment [months]	3.0 (0.0-10.4)	2.0 (0.0-8.1)	1.3 (0.0-12.0)	0.4087
Age at first intake of immunosuppressive treatment [years]	12.9 (10.3-14.6)	12.0 (9.2-14.7)	13.0 (9.1-14.3)	0.6795
Biological therapy	61 (48)	68 (34)	27 (34)	**0.0343**[2]
Number of biological agents	0 (0-1)	0 (0-1)	0 (0-1)	0.0612
Time-to-first dose of biological treatment [months]	14.0 (6.3-26.9)	12.6 (6.1-26.9)	22.7 (11.0-40.0)	0.2010
Age at first biological treatment	13.5 (10.7-15.3)	13.5 (10.7-15.5)	12.8 (10.4-15.1)	0.7249
Operative treatment	13 (10)	15 (8)	5 (6)	0.5588
Age at first surgery [years]	14.5 (13.1-16.3)	13.5 (10.4-14.8)	14.9 (14.6-15.7)	0.3673
Time-to-first surgery [months]	7.9 (0.4-39.8)	29.1 (16.7-43.0)	11.6 (3.0-27.0)	0.2214
**Hospitalisations (if duration ≥1 years)**				
Hospitalisations for relapse (per 1 year of the disease)	0.5 (0.3-0.9)	0.6 (0.2-1.0)	0.6 (0.3-1.1)	0.4678

Days of hospitalisation for relapse (per 1 year of the disease)	3.7 (1.6-8.3)	4.8 (1.2-8.0)	4.6 (1.7-7.4)	0.9635
Relapses from diagnosis (per 1 year of the disease)	0.5 (0.3-0.9)	0.6 (0.2-1.0)	0.6 (0.2-1.4)	0.8295
Severe relapses from diagnosis (per 1 year of the disease)	0.2 (0.0-0.5)	0.2 (0.0-0.6)	0.0 (0.0-0.4)	0.4427
**Concomitant diseases**	52 (41)	65 (33)	17 (21)	**0.0153**[3]
**Extraintestinal manifestations**	29 (23)	51 (26)	10 (13)	0.0542

[1] post hoc comparison: AA vs. AG p=0.0090 (Bonferroni and Holm)

[2] post hoc comparison: AA vs. AG p=0.0296 (Bonferroni and Holm)

[3] post hoc comparison: AA vs. GG p=0.0058 (Bonferroni and Holm)

Table S8. Association between *DMBT1* rs2981745 genotypes and IBD characteristics.

Variables<br>Median (IQR) or n (%)	CC	CT	TT	p value
**Selected biochemical parameters**				
CRP at diagnosis [mg/l]	6.6 (1.2-20.3)	3.9 (0.7-19.8)	9.8 (2.0-23.2)	0.3071
CRP at worst flare [mg/l]	6.4 (1.1-26.0)	4.8 (1.1-23.3)	14.3 (3.6-29.6)	0.2655
Albumin level at diagnosis [g/dl]	4.0 (3.6-4.3)	4.0 (3.7-4.4)	4.0 (3.6-4.4)	0.6535
Albumin level at worst flare [g/dl]	4.0 (3.6-4.4)	4.0 (3.6-4.4)	4.0 (3.6-4.3)	0.4418
**Treatment**				
Systemic steroids	126 (64)	105 (60)	22 (61)	0.7298
Number of courses of steroid treatment	1 (1-2)	1 (0-2)	1 (0-2)	0.5957
Immunosuppressive treatment	137 (70)	121 (70)	22 (61)	0.5461
Number of immunosuppressants	1 (0-1)	1 (0-1)	1 (0-1)	0.6719
Time-to-first dose of immunosuppressive treatment [months]	2.7 (0.0-9.2)	2.0 (0.0-7.6)	3.8 (0.0-24.3)	0.3640
Age at first intake of immunosuppressive treatment [years]	12.8 (9.6-14.7)	12.2 (9.2-14.7)	13.0 (9.2-14.4)	0.6821
Biological therapy	85 (43)	58 (33)	13 (36)	0.1352
Number of biological agents	0 (0-1)	0 (0-1)	0 (0-1)	0.1838
Time-to-first dose of biological treatment [months]	13.0 (6.1-25.2)	14.6 (8.0-29.7)	22.8 (7.6-40.2)	0.2593
Age at first biological treatment	13.6 (10.6-15.3)	13.2 (10.7-15.4)	13.6 (10.5-14.8)	0.8612
Operative treatment	20 (10)	9 (6)	4 (11)	0.1653
Age at first surgery [years]	14.0 (11.4-15.6)	14.7 (10.4-15.7)	14.7 (13.9-15.8)	0.7659
Time-to-first surgery [months]	10.0 (0.9-41.1)	28.9 (25.1-47.1)	7.3 (1.5-19.4)	0.8643
**Hospitalisations (if duration ≥1 years)**				
Hospitalisations for relapse (per 1 year of the disease)	0.6 (0.3-0.9)	0.6 (0.2-1.0)	0.6 (0.3-1.00)	0.8954

Days of hospitalisation for relapse (per 1 year of the disease)	4.7 (1.6-8.6)	4.3 (1.2-7.6)	3.8 (1.7-6.5)	0.5557
Relapses from diagnosis (per 1 year of the disease)	0.6 (0.3-1.0)	0.6 (0.2-1.0)	0.4 (0.0-1.1)	0.5129
Severe relapses from diagnosis (per 1 year of the disease)	0.2 (0.0-0.5)	0.1 (0.0-0.5)	0.0 (0.0-0.3)	**0.0352**[1]
**Concomitant diseases**	74 (38)	53 (30)	7 (19)	0.0638
**Extraintestinal manifestations**	48 (24)	38 (22)	4 (11)	0.2045

[1] post hoc comparison: CC vs. TT p=0.0170 (Bonferroni and Holm)

Table S9. Association between *SFTPD* rs2243639 genotypes and IBD characteristics.

Variables<br>Median (IQR) or n (%)	CC	CT	TT	p value
**Selected biochemical parameters**				
CRP at diagnosis [mg/l]	5.9 (0.7-23.2)	5.2 (1.1-19.8)	6.0 (1.0-23.6)	0.9325
CRP at worst flare [mg/l]	4.8 (1.1-31.5)	6.6 (1.6-21.3)	6.0 (1.3-25.0)	0.9873
Albumin level at diagnosis [g/dl]	4.1 (3.7-4.4)	4.0 (3.5-4.3)	4.0 (3.5-4.4)	0.2569
Albumin level at worst flare [g/dl]	4.0 (3.6-4.4)	4.0 (3.6-4.3)	4.0 (3.6-4.4)	0.9011
**Treatment**				
Systemic steroids	82 (58)	134 (66)	37 (60)	0.3012
Number of courses of steroid treatment	1 (0-2)	1 (1-2)	1 (0-2)	0.1245
Immunosuppressive treatment	95 (67)	141 (70)	44 (71)	0.8437
Number of immunosuppressants	1 (0-1)	1 (0-1)	1 (0-1)	0.8391
Time-to-first dose of immunosuppressive treatment [months]	2.0 (0.0-7.6)	2.0 (0.0-10.0)	3.4 (0.0-13.6)	0.4213
Age at first intake of immunosuppressive treatment [years]	12.6 (10.2-15.5)	12.3 (8.7-13.8)	13.4 (10.4-15.2)	**0.0476**[1]
Biological therapy	57 (40)	76 (37)	23 (37)	0.8322
Number of biological agents	0 (0-1)	0 (0-1)	0 (0-1)	0.7580
Time-to-first dose of biological treatment [months]	14.1 (4.2-29.1)	14.4 (8.0-26.7)	12.0 (6.3-27.0)	0.8003
Age at first biological treatment	13.7 (10.9-15.4)	13.2 (10.3-14.8)	14.5 (12.1-15.6)	0.3863
Operative treatment	12 (9)	15 (7)	6 (10)	0.8270
Age at first surgery [years]	14.4 (11.1-15.6)	14.0 (12.4-16.2)	14.1 (11.4-14.9)	0.9252
Time-to-first surgery [months]	20.1 (4.0-39.3)	25.1 (7.5-36.1)	8.3 (0.0-38.6)	0.9435
**Hospitalisations (if duration ≥1 years)**				
Hospitalisations for relapse (per 1 year of the disease)	0.6 (0.2-0.9)	0.6 (0.3-1.1)	0.5 (0.3-1.0)	0.4202

Days of hospitalisation for relapse (per 1 year of the disease)	3.7 (1.2-8.5)	4.8 (1.6-7.1)	4.6 (1.3-9.2)	0.6601
Relapses from diagnosis (per 1 year of the disease)	0.6 (0.2-0.9)	0.6 (0.3-1.1)	0.5 (0.2-1.2)	0.7207
Severe relapses from diagnosis (per 1 year of the disease)	0.1 (0.0-0.4)	0.2 (0.0-0.5)	0.2 (0.0-0.5)	0.8928
**Concomitant diseases**	43 (30)	68 (33)	23 (37)	0.6399
**Extraintestinal manifestations**	34 (24)	42 (21)	14 (23)	0.7511

[1] post hoc comparison: CC vs. CT p=0.0664 (Bonferroni and Holm)

Table S10. Association between *SFTPD* rs2243639 genotypes and CD characteristics.

Variables<br>Median (IQR) or n (%)	CC	CT	TT	p value
**Selected biochemical parameters**				
CRP at diagnosis [mg/l]	13.9 (2.0-36.0)	12.9 (2.3-23.0)	10.9 (1.5-24.9)	0.7675
CRP at worst flare [mg/l]	13.1 (3.0-45.7)	14.7 (3.1-28.5)	9.2 (3.5-25.0)	0.6068
Albumin level at diagnosis [g/dl]	4.0 (3.7-4.2)	3.8 (3.5-4.2)	4.0 (3.5-4.5)	0.5053
Albumin level at worst flare [g/dl]	3.9 (3.3-4.1)	3.9 (3.6-4.1)	4.0 (3.6-4.4)	0.3665
**Disease activity scales**				
PCDAI at diagnosis	35 (23-50)	30 (30-45)	33 (23-50)	0.8094
PCDAI at worst flare	43 (30-54)	43 (28-53)	40 (30-50)	0.8154
**Treatment**				
Systemic steroids	41 (55)	57 (54)	17 (50)	0.8919
Number of courses of steroid treatment	1 (0-2)	1 (0-2)	1 (0-1)	0.6835
Immunosuppressive treatment	60 (80)	82 (78)	26 (76)	0.9083
Number of immunosuppressants	1 (1-1)	1 (1-1)	1 (1-1)	0.6243
Time-to-first dose of immunosuppressive treatment [months]	2.0 (0.0-7.1)	1.0 (0.0-9.6)	2.8 (0.0-17.7)	0.4798
Age at first intake of immunosuppressive treatment [years]	12.4 (10.6-15.3)	12.8 (9.8-13.7)	13.7 (11.4-15.3)	0.1852
Biological therapy	39 (52)	53 (50)	15 (44)	0.7418
Number of biological agents	1 (0-1)	1 (0-1)	0 (0-1)	0.4784
Time-to-first dose of biological treatment [months]	14.5 (4.2-32.0)	14.1 (6.2-25.2)	11.0 (6.7-30.9)	0.9401
Age at first biological treatment	14.1 (12.0-15.4)	13.5 (10.8-14.9)	14.7 (12.6-15.1)	0.4542
Operative treatment	9 (12)	15 (14)	5 (15)	0.8869
Age at first surgery [years]	14.4 (14.0-15.3)	13.9 (12.8-16.0)	14.6 (13.5-14.9)	0.8696
Time-to-first surgery [months]	23.3 (6.7-49.2)	25.1 (7.7-32.6)	0.0 (0.0-38.6)	0.4623
**Hospitalisations (if duration ≥1 years)**				

Hospitalisations for relapse (per 1 year of the disease)	0.5 (0.2-0.7)	0.5 (0.3-1.0)	0.4 (0.2-0.6)	0.2846
Days of hospitalisation for relapse (per 1 year of the disease)	3.7 (1.2-7.4)	4.7 (1.6-6.7)	2.7 (0.5-8.3)	0.7614
Relapses from diagnosis (per 1 year of the disease)	0.5 (0.2-0.8)	0.5 (0.2-1.0)	0.4 (0.2-0.9)	0.7608
Severe relapses from diagnosis (per 1 year of the disease)	0.2 (0.0-0.4)	0.2 (0.0-0.5)	0.3 (0.0-0.5)	0.8004
**Concomitant diseases**	23 (31)	27 (26)	14 (41)	0.2275
**Extraintestinal manifestations**	18 (24)	28 (27)	7 (21)	0.7612
